# Overexpression of the *Salix matsudana SmAP2-17* gene improves *Arabidopsis* salinity tolerance by enhancing the expression of *SOS3* and *ABI5*

**DOI:** 10.1186/s12870-022-03487-y

**Published:** 2022-03-07

**Authors:** Yanhong Chen, Yuanhao Dai, Yixin Li, Jie Yang, Yuna Jiang, Guoyuan Liu, Chunmei Yu, Fei Zhong, Bolin Lian, Jian Zhang

**Affiliations:** Key Lab of Landscape Plant Genetics and Breeding, School of Life Science, Nantong University, Nantong, Jiangsu Province China

**Keywords:** *Salix matsudana*, AP2/ERF, Salt tolerance, *Arabidopsis*, Overexpression

## Abstract

**Background:**

*Salix matsudana* (Koidz.) is a widely planted ornamental allotetraploid tree species. Genetic engineering can be used to enhance the tolerance of this species to soil salinization, endowing varieties with the ability to grow along coastlines, thereby mitigating afforestation and protecting the environment. The AP2/ERF family of transcription factors (TFs) plays multidimensional roles in plant biotic/abiotic stress tolerance and plant development. In this study, we cloned the *SmAP2-17* gene and performed functional analysis of its role in salt tolerance. This study aims to identify key genes for future breeding of stress-resistant varieties of *Salix matsudana*.

**Results:**

*SmAP2-17* was predicted to be a homolog of AP2-like ethylene-responsive transcription factor ANT isoform X2 from *Arabidopsis*, with a predicted ORF of 2058 bp encoding an estimated protein of 685 amino acids containing two conserved AP2 domains (PF00847.20). *SmAP2-17* had a constitutive expression pattern and was localized to the nucleus. The overexpression of the native *SmAP2-17* CDS sequence in *Arabidopsis* did not increase salt tolerance because of the reduced expression level of ectopic *SmAP2-17*, potentially caused by salt-induced RNAi. Transgenic lines with high expression of optimized *SmAP2-17* CDS under salt stress showed enhanced tolerance to salt. Moreover, the expression of general stress marker genes and important salt stress signaling genes, including *RD29A*, *ABI5*, SOS3, *AtHKT1,* and *RBohF,* were upregulated in *SmAP2-17*-overexpressed lines, with expression levels consistent with that of *SmAP2-17* or optimized *SmAP2-17*. Promoter activity analysis using dual luciferase analysis showed that SmAP2-17 could bind the promoters of *SOS3* and *ABI5* to activate their expression, which plays a key role in regulating salt tolerance.

**Conclusions:**

The *SmAP2-17* gene isolated from *Salix matsudana* (Koidz.) is a positive regulator that improves the resistance of transgenic plants to salt stress by upregulating *SOS3* and *ABI5* genes. This study provides a potential functional gene resource for future generation of salt-resistant *Salix* lines by genetic engineering.

**Supplementary Information:**

The online version contains supplementary material available at 10.1186/s12870-022-03487-y.

## Background

The APETALA2/ethylene responsive factor (AP2/ERF) family transcription factors (TFs) is a plant-specific gene family with many members, which are classified into APETALA2(AP2), RELATED TO ABSCISIC ACID INSENSITIVE3/VIVIPAROUS 1 (RAV), DEHYDRATION-RESPONSIVE ELEMENT BINDING proteins (DREBs), ETHYLENE RESPONSIVE FACTORS (ERFs), and Soloist subfamilies, according to the number or structure of AP2 and other conserved domains [1, 2]. AP2/ERF family TFs play important and multidimensional roles in plant biotic/abiotic stress tolerance and plant growth, differentiation, and development [3–8]. DREB members extensively participate in the response and regulation of abiotic stress tolerance, such as cold, drought, heat, and salt tolerance, by directly regulating the expression of stress-responsive genes [5, 9]. In addition to genes from *Arabidopsis*, many studies have illustrated the function of DREB genes from other plant species, such as maize, wheat, and soybean, by overexpressing these genes in model plants [10–16]. Members of the ERF subfamily are also involved in abiotic stress responses, and members of the ERF-VII subfamily from *Arabidopsis* and rice have been verified to play major roles in flooding and submergence tolerance (hypoxia stress) [17–19]. *Arabidopsis* AP2s play a central role in developmental processes, including axillary floral meristem development, gynoecium development, shoot regeneration, brace root development, internode elongation, and trichome formation [20–25]. Members of the AP2 subfamilies have also been reported to mediate diverse abiotic stress responses. AINTEGUMENTA (ANT) is an example of this. In addition to controlling organ cell number and size, ANT also negatively regulates salt tolerance by repressing SOS3-LIKE CALCIUM BINDING PROTEIN 8 (SCABP8/CBL10), a putative Ca^2+^ sensor that protects *Arabidopsis* shoots against salt stress and maintains ion homeostasis [26].

*Salix matsudana* Koidz., an allotetraploid member of *Salicaceae*, is an important and widely planted ornamental tree species with strong and wide adaptability to diverse environmental stresses, including salinity [27, 28]. The genome of *S. matsudana* was recently sequenced, and the family members and classification of the *SmAP2/ERF* gene family were identified and their expression patterns under salt stress were analyzed [28, 29]. From the sequencing results, 364 SmAP2/ERF members were identified and named from the *S. matsudana* genome and classified into four major subfamilies: AP2 (55 members), ERF (166 members), DREB (135 members), and RAV (6 members) [29]. The expression levels of 4 genes from the AP2 subgroup, 10 genes from the DREB subgroup, and 13 genes from the ERF subgroup were strongly induced by salt stress [29]. Considering the important roles of AP2/ERF family TFs in stress tolerance, there is an urgent need to uncover the function of the *SmAP2/ERF* genes, especially those genes with induced expression patterns under stress. These researches will promote the application of these genes in molecular assisted breeding of *Salix* plants. However, because of the difficulties in genetic transformation and the unavailability of genomic sequences, the functional characterization of SmAP2/ERF members remains largely unexplored in *Salix* and other woody plant species. In the present study, a putative AP2 family gene, *SmAP2-17*, whose expression was induced under salt stress, was isolated and cloned from *Salix matsudana*. To determine the function of *SmAP2-17*, this gene was transformed into *Arabidopsis* via *Agrobacterium*-mediated transformation to evaluate its role in tolerance to salt stress and uncover its downstream genes.

## Results

### Isolation and bioinformatics analysis of SmAP2-17 and its homolog proteins

**Fig. 1:**
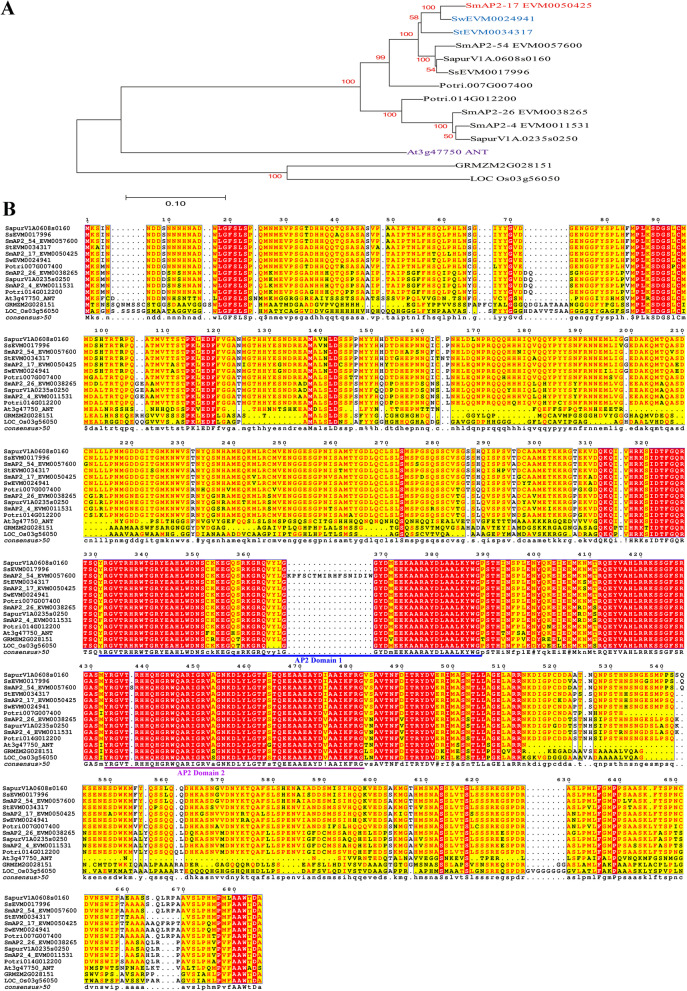
Phylogenetic analysis and sequence alignment of SmAP2-17 and its homologs. **a** Phylogenetic analysis of the SmAP2-17 protein with its orthologous genes in other plant species. The phylogenetic tree was constructed using the neighbor-joining method with 1000 bootstrap replicates in MEGA 7.0 software. **b** Alignment of the SmAP2-17 protein with its orthologous genes in *Salix*, *Populus*, maize and *Arabidopsis*. There are two AP2 domains in the proteins, which were labeled with blue and pink lines, respectively. ANT is an *Arabidopsis* protein, whose gene locus is At3g47750. SapurV1A.0608s0160.1 and Potri.007G007400.1 come from *Salix purpurea* and *Populus trichocarpa*, respectively. GRMZM2G028151_T01 and LOC_Os03g56050.1 are proteins from maize and rice. Proteins with Sw, Sm, St, and Ss denote proteins that belong to *Salix wilsonii*, *Salix matsudana*, *Salix integra* and *Salix suchowensis*, respectively

The coding sequence of *SmAP2-17* was 2058 bp in length and encoded a protein comprised of 685 amino acids with a molecular mass of 76.3 kDa and an isoelectric point of 6.45. In the genome annotation, SmAP2-17 was predicted to be a homolog of AP2-like ethylene-responsive transcription factor ANT isoform X2. SmAP2-17 shares an identity of 44.65% with *Arabidopsis* ANT protein. Phylogenetic analysis showed that SmAP2-17 was closely related to proteins from *Salix* and *Populus*, such as SwEVM0024941.1 (96% identity), SapurV1A.0608s0160.1 (92.81% identity), and Potri.007G007400.1 (87.01% identity) from *Salix wilsonii*, *Salix purpurea*, and *Populus trichocarpa*, respectively (Fig. 1a). Other homologs shared a lower identity, in particular GRMZM2G028151_T01 and LOC_Os03g56050.1 from maize and rice, respectively. Multiple sequence alignment analysis showed that these proteins shared two conserved AP2/ERF domains (Fig. 1b). The protein sequences of SmAP2-17 and its homologs are listed in File S1.

### Analysis of promoter elements of *SmAP2-17* and its homolog genes

**Fig. 2:**
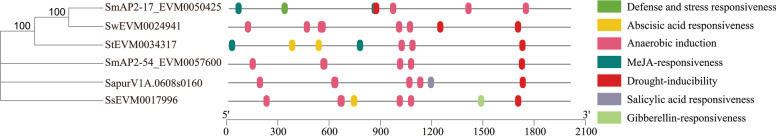
Cis-acting regulatory elements related to the stress and hormone response in the promoter regions of *SmAP2-17* and its homolog genes from *Salix*. The cis-acting elements in the promoter of *SmAP2-17* and its five homolog genes from *Salix* were predicted using the online PlantCARE tool and illustrated using TBtools software. Detailed information is provided in Table S1

The 2000-bp promoter sequences of* SmAP2-17* and its five homologs in *Salix* were isolated (File S2) and cis-element motif analysis was performed using the PlantCARE database (http://bioinformatics.psb.ugent.be/webtools/plantcare/html/). The anaerobic induction motif had the highest number and was present in the promoter region of all genes. The drought-inducibility motif also appeared in all genes, but most genes had only one (the only exception being *SwEVM0024941*, having two). Apart from the anaerobic induction motif and drought-inducibility motif, the abscisic acid responsiveness motif, defense and stress responsiveness motif, and MeJA responsiveness are also present in the promoter region of *SmAP2-17* (the position of the abscisic acid responsiveness motif and MeJA-responsiveness motif almost overlapped, Table S1). The abscisic acid responsiveness motif was also found in *StEVM0034317* and *SsEVM0017996*. The salicylic acid responsiveness motif and gibberellin-responsiveness motif were only found in *SapurV1A.0608s0160* and *SsEVM0017996*, respectively (Fig. 2).

### Expression pattern and subcellular location

**Fig. 3:**
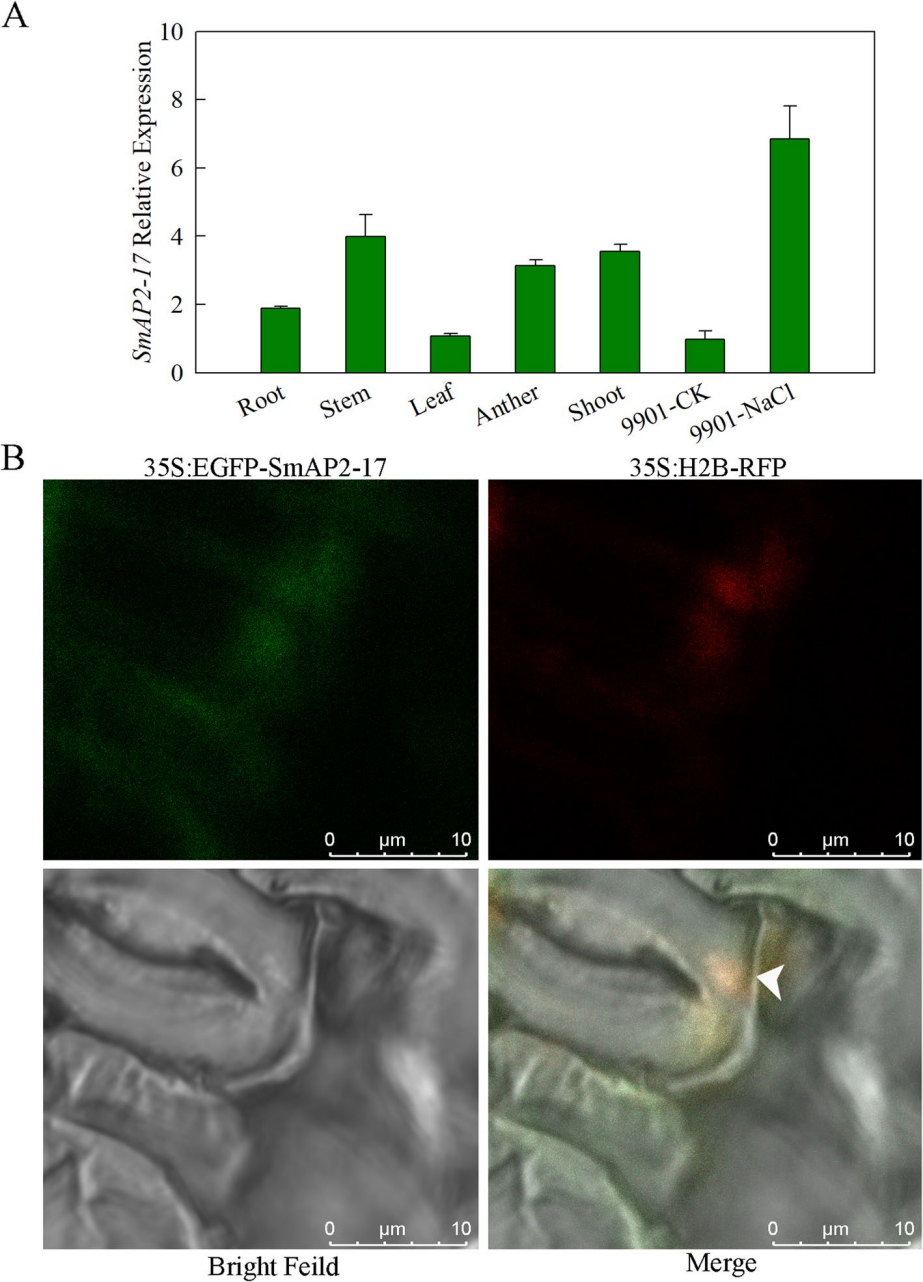
Expression pattern and subcellular localization of SmAP2-17 protein. **a** Expression patterns of *SmAP2-17* in the roots, stems, leaves, anthers, and shoots of *S. matsudana* and under salt stress were measured using qRT-PCR. **b** Subcellular localization of the SmAP2-17 protein. The 35S:EGFP-SmAP2-17 fusion construct and the nucleus localization marker 35S:H_2_B-RFP construct were co-transformed into tobacco epidermal leaves. The arrowhead indicates the merged signal (yellow) with EGFP (green) and RFP (red) co-located in the nucleus. Scale bar, 10 μm

Gene expression patterns can provide useful clues to gene function. To study the expression profiles of *SmAP2-17*, the transcripts of *SmAP2-17* across different tissues and under salt stress were examined using qRT-PCR. In different tissues (root, stem, leaf, shoot, and anther) of *S. matsudana*, the highest accumulation of *SmAP2-17* was observed in the stems and shoots, followed by the roots and anther, while low expression levels were detected in the leaves. Salt stress induced the expression of *SmAP2-17*, with an expression level that was eightfold higher than normal conditions (Fig. 3a).

The subcellular localization of SmAP2-17 was examined to gain useful insights into its molecular function. WoLF PSORT (http://www.genscript.com/wolf-psort.html) predicted that SmAP2-17 resides in the nucleus. To confirm this prediction, the coding sequence of *SmAP2-17* was fused to the C-terminus of green fluorescent protein (GFP) driven by the cauliflower mosaic virus 35S promoter, and the construct was transiently expressed in tobacco leaf epidermal cells. The expression of the red fluorescent protein (RFP) fusion histone protein, H_2_B-RFP, was used as a nuclear localization marker. As shown in Fig. 3b, a yellow fluorescence signal from the co-localization of SmAP2-17-GFP construct (green) and H_2_B-RFP (red) was observed in the nucleus of leaf stomata guard cells. This finding indicates that SmAP2-17 was localized in the nucleus (Fig. 3b).

### *SmAP2-17* did not confer transgenic *Arabidopsis* tolerance to salt stress due to salt-induced gene silencing

**Fig. 4:**
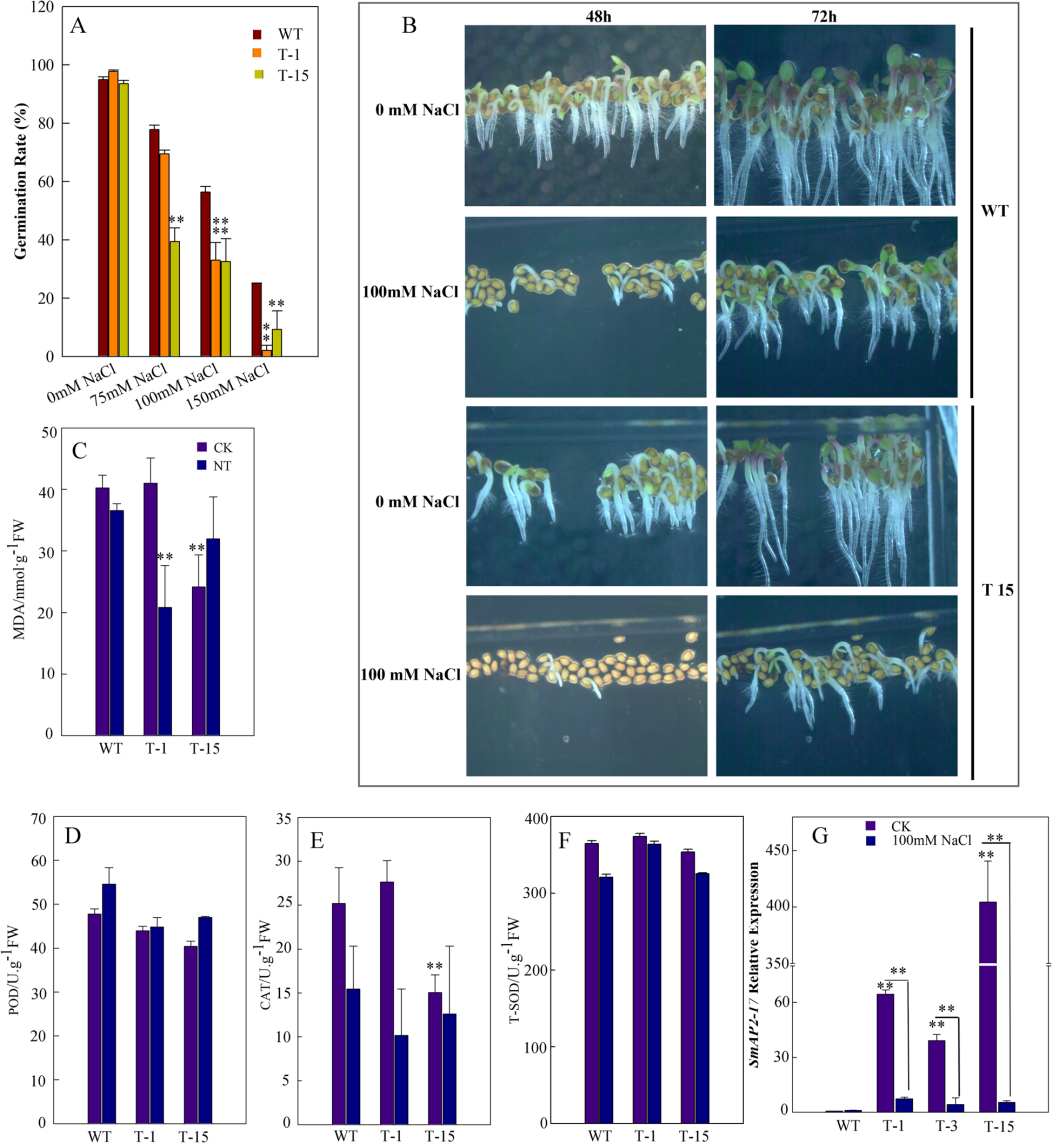
*SmAP2-17*-overexpression transgenic lines did not show increased tolerance to salt stress. **a** *SmAP2-17*-overexpression transgenic line T-15 had a delayed germination phenotype under salt stress compared to the WT (wild-type) at 48 h and 72 h after sowing. **b** Statistical analysis of the germination rate of WT and transgenic lines T-15 and T-1 at 72 h after sowing under different concentrations of NaCl (75 mM NaCl, 100 mM NaCl, and 150 mM NaCl). **c** qRT-PCR analysis of the expression levels of *SmAP2-17* in WT and transgenic lines T-1 and T-15 under normal conditions and after treatment with 100 mM NaCl. **c-f** Physiological indices of transgenic and WT *Arabidopsis* seedlings under normal and salt stress conditions. The MDA content (**c**) and antioxidant enzyme activities, including peroxidase (**d**), catalase (**e**), and superoxide dismutase (**f**), were measured in WT and transgenic lines T-15 and T-1 under normal and salt stress conditions. Two-week-old seedlings were exposed to 200 mM NaCl for 4 d before the measurements. **g** qRT-PCR analysis of the expression levels of *SmAP2-17* in WT and transgenic lines T-1 T-3, and T-15 under normal and salt stress (100 mM) conditions. Data represent the mean of three replicates, and the error bars indicate the SD; *P < 0.05 and **P < 0.01 by Student’s t-test

To characterize the role of SmAP2-17 in salt tolerance, the construct containing the *SmAP2-17* coding sequence (CDS) driven by the 35S enhancer was transformed into *Arabidopsis*. After screening and molecular identification, two transgenic lines were used to perform different treatments of NaCl on 1/2 MS medium to determine the germination rates of *Arabidopsis*. Upon salt treatment using 75 mM NaCl, 100 mM NaCl, and 150 mM NaCl, the germination rates of the T1 and T15 transgenic lines at 72 h were significantly lower than those of the wild-type (WT) (Fig. 4a). The delayed germination phenotype could be observed clearly in photographs (Fig. 4b), which showed germination and growth phenotypes of WT and T15 at 48 h and 72 h after planting the seeds on 1/2 MS medium or 1/2 MS medium supplemented with 100 mM NaCl (Fig. 4b).

Salt stress can induce oxidative stress through the generation and accumulation of reactive oxygen species (ROS). Antioxidant enzymes, such as peroxidase (POD), superoxide dismutase (SOD), and catalase (CAT), play key roles in scavenging ROS and protecting plants from oxidative damage [30]. The malondialdehyde (MDA) content reflects the degree of lipid peroxidation and indirectly reflects the degree of cell damage [30]. The activities of these antioxidant enzymes and MDA content were also measured in the *SmAP2-17*-overexpression and WT plants. The results showed that there was no substantial difference in the activities of POD and SOD under normal conditions and after salt stress treatment. The MDA content was lower in T-15 under normal conditions and in T-1 under salt treatment. CAT activity was lower in T-15 than in WT and T-1, but no marked difference was observed under salt stress (Fig. 4c-f).

The data from lower CAT enzyme activity and higher MDA content were consistent with the salt sensitive phenotype of the overexpression plants, which did not exhibit increased salt tolerance. To determine the role of *SmAP2-17*, we detected the expression level of *SmAP2-17* in overexpression plants after salt stress, and found that the expression level of *SmAP2-17* decreased sharply in all lines (Fig. 4g). Considering that the higher level of *SmAP2-17* expression in the *SmAP2-17*-overexpression was silenced after salt stress, we proposed that *SmAP2-17* mRNA may be the target of a putative salt-induced miRNA molecule. Recently, a large number of abiotic stress-induced miRNAs, including the salt-induced miRNA gene ath-MIR167d, have been reported [31]. The secondary complementary structure of *SmAP2-17* mRNA and a salt-induced miRNA ath-MIR167d suggest that SmAP2-17 mRNA may be digested through ath-MIR167d mediated RNAi process (Fig. S1).

### Phenotypic and physiology analysis of codon-optimized *SmAP2-17* gene overexpression transgenic lines

**Fig. 5:**
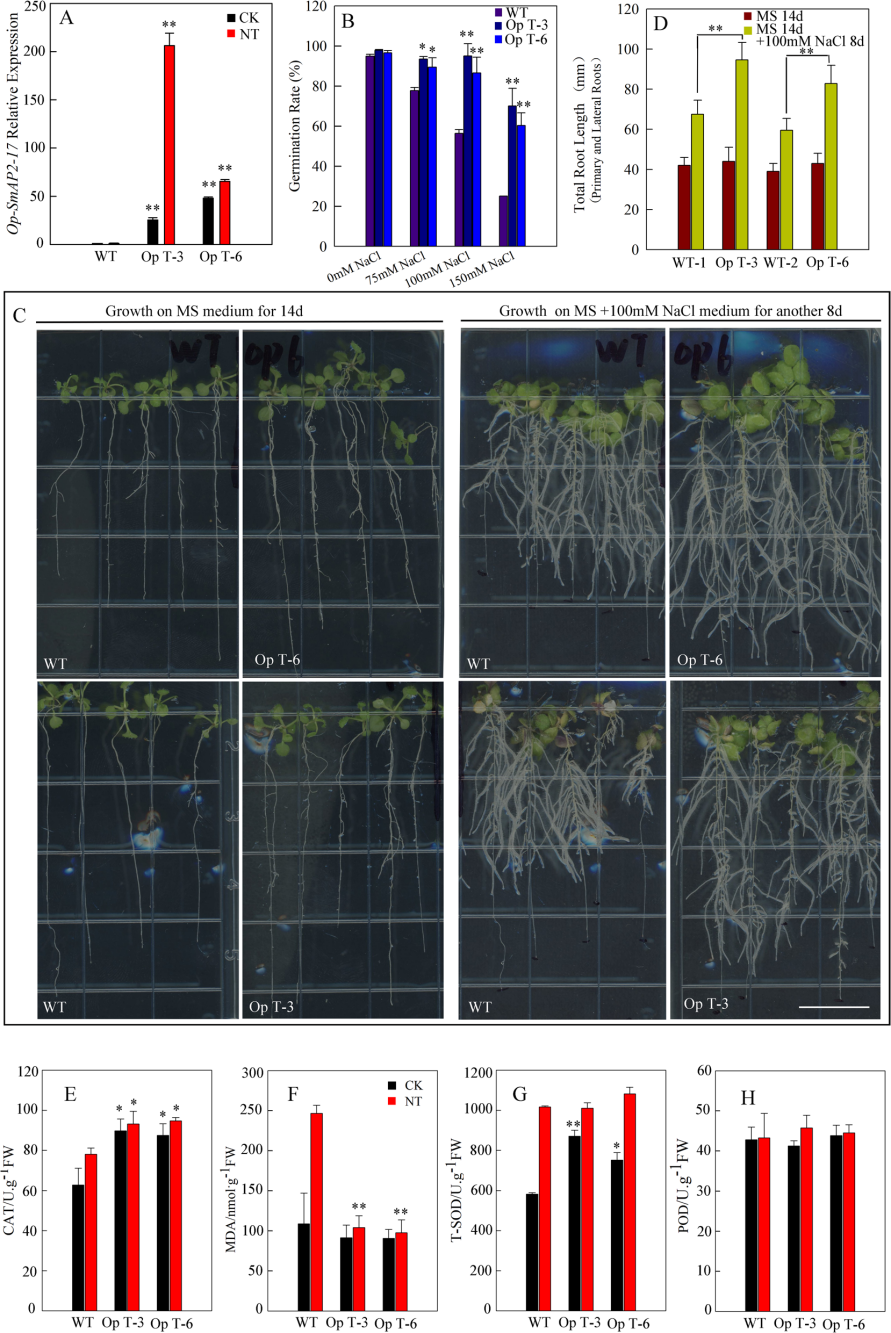
Effects of codon-optimized *SmAP2-17*-overexpression on salt stress tolerance in transgenic *Arabidopsis*. **a** qRT-PCR analysis of the expression levels of codon-optimized *SmAP2-17-*overexpression in WT and transgenic lines Op T-3 and Op T-6 under normal and salt stress (100 mM) conditions. **b** Statistical analysis of the germination rate of the WT and transgenic lines Op T-3 and Op T-6 at 72 h after sowing under different concentrations of NaCl (75 mM NaCl, 100 mM NaCl, and 150 mM NaCl). **c** Transgenic lines Op T-3 and Op T-6 had more and longer lateral roots than the WT. **d** Statistical comparison of the total root length (primary root + [lateral root number × average length of lateral roots]) between WT and transgenic lines Op T-3 and Op T-6. **e**–**h** Physiological indices of transgenic and WT *Arabidopsis* seedlings under normal and stress conditions. The MDA content (**e**) and antioxidant enzyme activities, including peroxidase (**f**), catalase (**g**), and superoxide dismutase (**h**), were measured in the WT and transgenic lines Op T-3 and Op T-6 under normal and salt stress conditions. Two-week-old seedlings were exposed to 200 mM NaCl for 4 d before the measurements. Data represent the mean of three replicates, and the error bars indicate the SD; *P < 0.05 and **P < 0.01 by Student’s t-test

Based on our assumption, we optimized the *SmAP2-17* mRNA codons of the ath-MIR167d binding site and placed the optimized CDS behind the 35S enhancer in the plant binary vector. The new *SmAP2-17*-overexpression construct was named Op- *SmAP2-17* and transformed into *Arabidopsis* via the floral dip method. The Op- *SmAP2-17*-overexpression transgenic lines were screened and verified following the same protocol as the native *SmAP2-17* gene. First, the expression level of Op- *SmAP2-17* was detected in the WT and Op-*SmAP2-17*-overexpression transgenic lines Op T-3 and Op T-6 in 1/2 MS medium and medium containing 100 mM NaCl. The qRT-PCR results showed that salt stress did not decrease the expression level of Op-*SmAP2-17* mRNA in Op T-3 and Op T-6 lines. In contrast, the expression level in Op T-3 increased by approximately tenfold when compared with that in WT control (Fig. 5a).

The *Op-SmAP2-17-*overexpression transgenic lines Op T-3 and Op T-6 were subjected to 72 h of germination. The results showed that, contrary to the germination rate of native SmAP2-17 transgenic lines, Op T-3 and Op T-6 had higher germination rates than the WT when treated with 75 mM NaCl, 100 mM NaCl, and 150 mM NaCl (Fig. 5b). A higher difference was found after treatment with 150 mM NaCl, the germination rate of WT decreased to about 20%, while about 60% of the Op T-3 and Op T-6 seeds still could germinate (Fig. 5b). To further evaluate the salt tolerance phenotype of Op T-3 and Op T-6, the growth of these plants was assessed on MS medium containing 100 mM NaCl. After 14 days of growth on normal MS medium, the seedlings of the WT, Op T-3, and Op T-6 lines were transplanted to MS medium containing 100 mM NaCl and grown for another eight days. The results showed that after 8 days of treatment with 100 mM NaCl, the seedlings of Op T-3 and Op T-6 grew more and longer lateral roots than the WT (Fig. 5c). The number and length of the primary and lateral roots were counted, and the total root length was calculated (primary root + [lateral root number × average length of lateral roots]) for comparison (Fig. 5d). The total root length of Op T-3 and Op T-6 was found to increase by 40–50% compared to that of the WT (Fig. 5d).

With regards to the four physiological indexes, the CAT enzyme content in the two Op lines was higher than that in the WT after treatment with 200 mM NaCl (Fig. 5e). By contrast, the MDA content was lower than in the WT under the same conditions (Fig. 5f). The SOD enzyme content in the two Op lines was higher than that in the WT under normal conditions, whereas no difference was found in these lines under salt stress (Fig. 5g). There was no difference in the POD content under both the normal and salt stress conditions (Fig. 5h).

### Analysis of gene expression pattern of general stress marker genes and salt stress-related genes in *SmAP2-17-*overexpression transgenic lines

Plants can activate many transcriptional and physiological processes in order to grow and survive under conditions of salt and abiotic stress. Several genes have regulatory roles and positive effects on abiotic stress tolerance, including stress-responsive marker genes (*DREB2A*, *RD20*, *ABI5*, *RD29A*, *ATGolS2*, *ABF3*, *AtMYB2*, and *RD29B*) and salinity-responsive genes (*AtNXH1*, *AtNXH2*, *AtHKT1*, *RBohF*, and *AtSOS3*), which play important roles in helping plants to cope with and survive under conditions of salt stress [32–36].

**Fig. 6:**
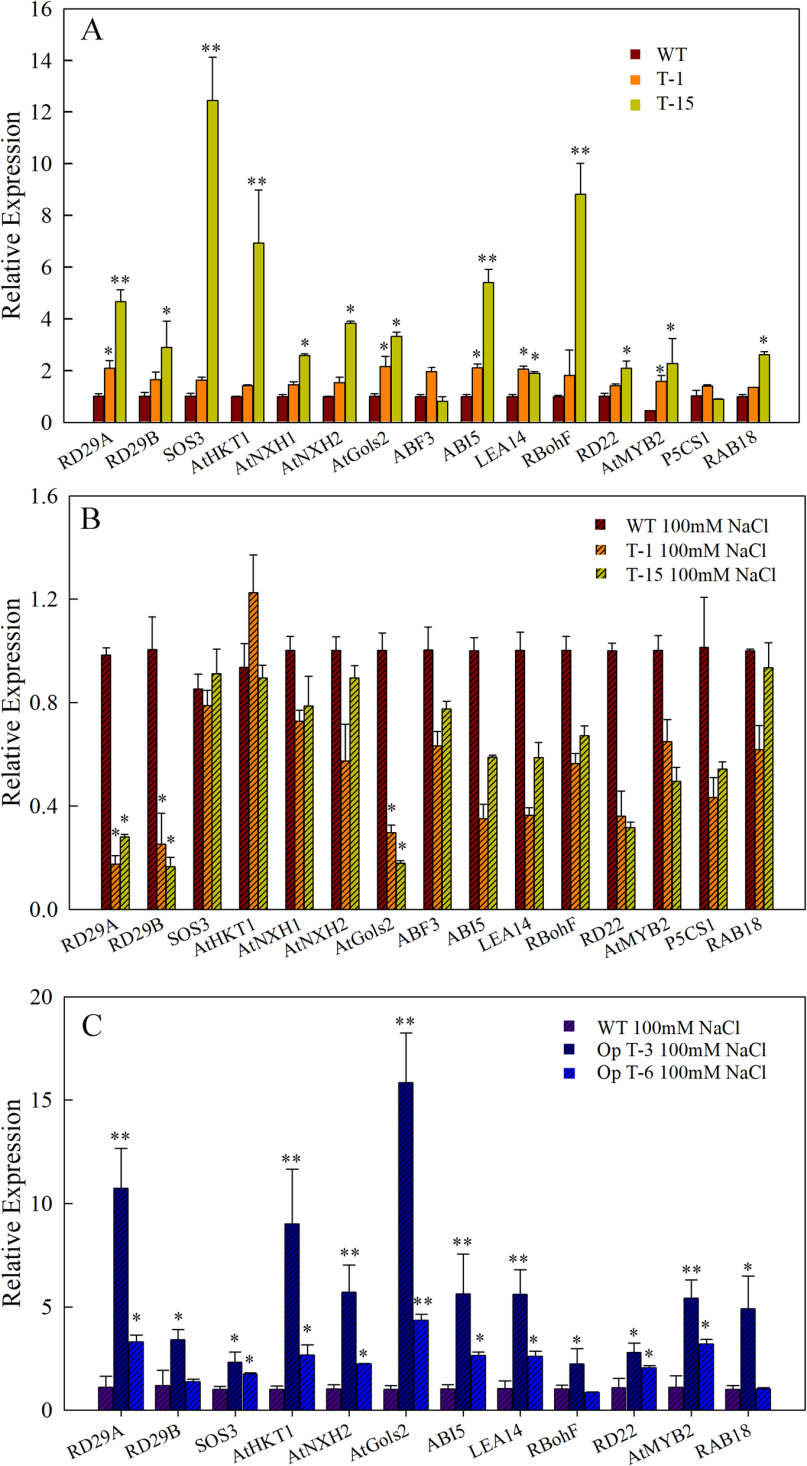
Relative expression levels of stress responsive marker genes and salinity responsive genes in WT and transgenic lines under normal conditions and after treatment with NaCl. **a** Relative expression levels of 15 genes in the WT and transgenic lines T-1 and T-15 under normal conditions. **b** Relative expression levels of 15 genes in the WT and transgenic lines T-1 and T-15 treated with 200 mM NaCl for 24 h. **c** Relative expression levels of 12 genes in the WT and transgenic lines Op T-3 and Op T-6 treated with 200 mM NaCl for 24 h. Data represent the mean ± SD of three biological replicates. *P < 0.05 and **P < 0.01 by Student’s t-test

To further explore the downstream genes regulated by SmAP2-17 under salt stress, the expression patterns of stress/salt-responsive genes were evaluated by qRT-PCR. In the native *SmAP2-17*-overexpression transgenic lines, under normal conditions, many genes were found to be upregulated, especially in T-15, which expressed *SmAP2-17* at extremely high levels. The expression levels of 13 out of 15 genes we detected were upregulated by over two-fold as compared to those in the WT (Fig. 6a). The top five genes with the highest increase in expression were *RD29A*, *ABI5*, *SOS3*, *AtHKT1*, and *RBohF*. In the transgenic line T-15, the expression level of SOS3 was over 12-fold higher than that in the WT (Fig. 6a). Interestingly, these five genes were induced with much higher expression levels than those in T-1, and the expression level of these genes was consistent with the higher expression of *SmAP2-17* in T-15 (Fig. 6a).

After treatment with 100 mM NaCl, the expression patterns of these genes in T-1 and T-15 were also detected by qRT-PCR. Following the gene silencing of *SmAP2-17* after salt stress, the expression of all genes detected was inhibited with expression levels that were lower or the same under the salt stress treatment (Fig. 6b).

In the Op T-3 and Op T-6 overexpression transgenic lines with optimized codons of *SmAP2-17* CDS, the expression patterns of 12 genes were examined after salt stress. The results showed that these genes in T-1 and T-15 were not inhibited in the Op T-3 and Op T-6 lines. All of the genes had a higher expression level than that in the WT, and the expression level of these genes was consistent with the higher expression level of Op-SmAP2-17 in Op T-3 (Fig. 6c).

### Promoter motif analysis on putative downstream gene of *SmAP2-17*

**Fig. 7:**
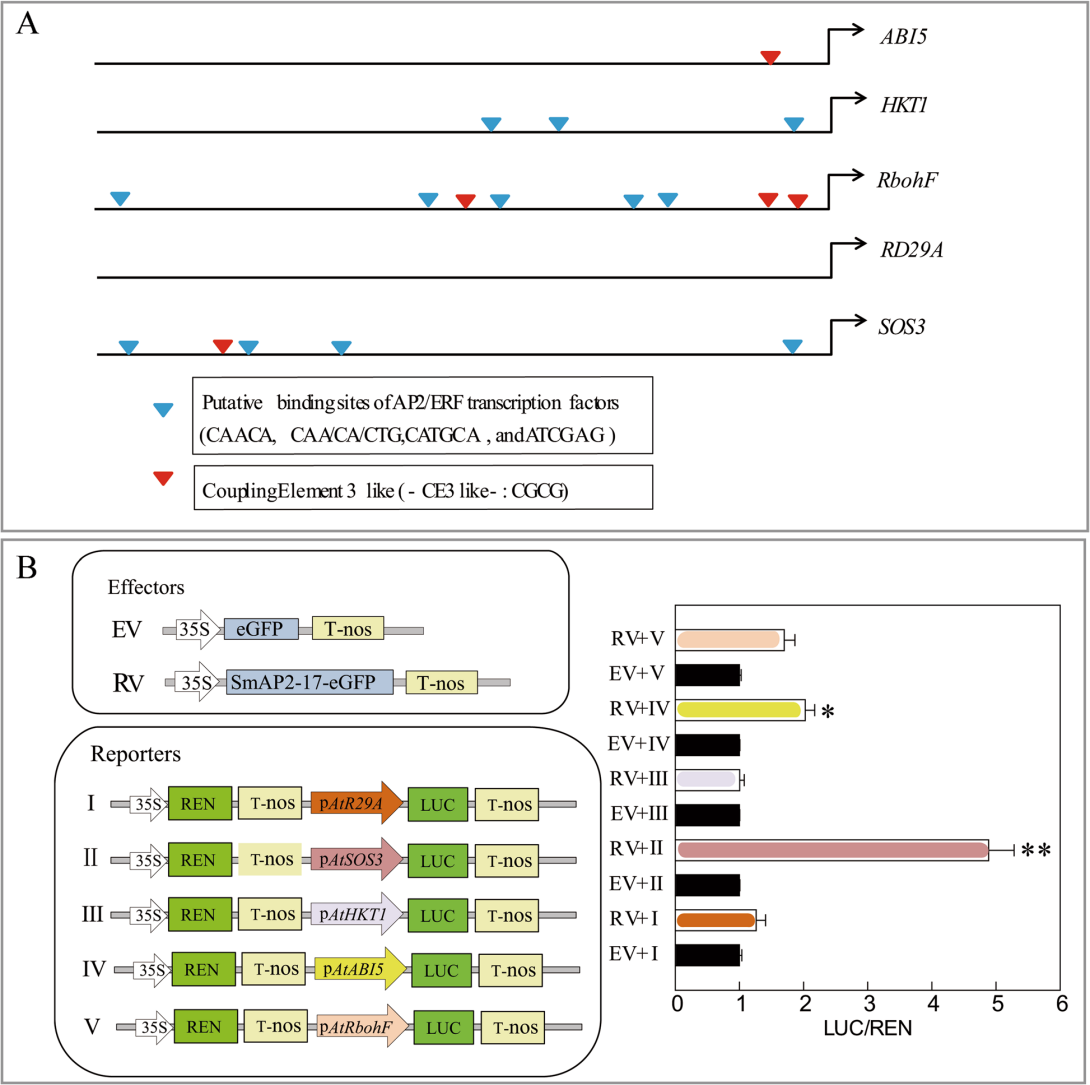
*SOS3* and *ABI5* expression is activated in the presence of *SmAP2-17* as measured by the luciferase reporter assays. **a** Schematic diagrams of the putative AP2/ERF binding elements in the 2000-bp promoter region of the five candidate downstream genes. **b** Left panel: Schematic diagrams of effector and reporter plasmids used in transient transactivation assays: top, schematic diagrams of control (EV, empty vector) and effector (RV, recombination vector); bottom, schematic diagrams of reporter constructs. The promoter sequences from five candidate downstream genes were cloned upstream of the *LUC* reporter gene. The *REN* located downstream of the 35S promoter was used as an internal control. Right panel: The results showed that five candidate downstream genes, *SOS3* and *ABI5,* were regulated by SmAP2-17. Relative activity was calculated as *LUC*/*REN*, but was normalized to the reporter only. Data represent the mean ± SD of three biological replicates. *P < 0.05 and **P < 0.01 by Student’s t-test

Based on the expression pattern of these genes, consistent with the expression level of *SmAP2-17* in T-15 and Op-SmAP2-17 in OpT-3, we selected five genes as putative downstream genes of *SmAP2-17*: *ABI5*, *HKT1*, *RbohF*, *RD29A*, and *SOS3*. We downloaded the 2000-bp promoter sequence of the five genes and performed motif analysis using the PlantCARE platform. The putative binding site motifs of AP2/ERF transcription factors (including CAACA, CAA/CA/CTG, CATGCA, and ATCGAG) and coupling element 3-like (CE3-like) (CGCG) were identified in the promoters of these five genes (Fig. 7a). No AP2 binding and CE3-like motifs were found in the *RD29A* gene promoter, while only one CE3-like motif was identified in the *ABI5* gene promoter and three AP2 binding motifs were present on the *HKT1* gene promoter. *RbohF* had the highest number of these motifs, including five AP2 binding motifs and three CE3-like motifs. The last one gene, *SOS3*, had four AP2 binding motifs and one CE3-like motif (Fig. 7a).

### SmAP2-17 binds the promoter region of *SOS3* and *ABI5* and drives the expression of the *Luc* gene

Next, a dual luciferase reporter assay was used to determine whether these five genes were the direct downstream genes of *SmAP2-17*. The 2000-bp promoter sequences of *ABI5*, *HKT1*, *RbohF*, *RD29A,* and *SOS3* were cloned upstream of the *LUC* gene in the pGreenII 0800-LUC vector. *REN* was used as an internal control, and a *SmAP2-17*-overexpression vector was also constructed. The *SmAP2-17*-overexpression vector construct and each gene promoter::*LUC* construct were co-transformed into tobacco leaf cells via agrobacteria-mediated transformation. The signal intensities of *LUC* and *REN* were detected, and the *LUC*/*REN* ratios were calculated. The results showed that from the five genes analyzed, SmAP2-17 could bind to the promoter region of *SOS3* and *ABI5* and activate the expression of the downstream *LUC* gene (Fig. 7b). In the ectopic system, these results showed that SmAP2-17 may directly bind to the upstream regulatory region and upregulate the expression levels of *SOS3* and *ABI5*, thereby enhancing the salt tolerance of the *SmAP2-17*-overexpression transgenic lines.

## Discussion

The APETALA2/ethylene response factor (AP2/ERF) transcription factor (TF) is a superfamily of plant species that plays multiple roles in plant growth and development, stress tolerance, and the integration of phytohormonal signals [3]. Since the first AP2/ERF TF AP2 was identified in *Arabidopsis* as a controller in flower and seed development, several genes of the DREB and ERF subfamily have been explored for their molecular roles and mechanisms, particularly in biotic and abiotic stresses [9, 11, 14, 37–39]. Previous studies on the function of AP2/ERF have mainly focused on model plants and crops, and some studies have explored the *AP2/ERF* genes of trees, mainly from *Populus* and *Broussonetia papyrifera* [40–43]. *Salix matsudana* (Koidz.) is an important and widely cultivated ornamental tree species [28]. As a result of the genome-wide analysis of AP2/ERF family members of *Salix matsudana* (Koidz.), the differential expression patterns of some *SmAP2/ERF* genes under salt stress have been investigated [29]. However, little is known about the functional roles of *SmAP2/ERF* and their mechanisms in *Salix*. Transferring a single gene encoding stress-tolerance transcription factors into plants could improve plant drought, salt, and freezing tolerance. Therefore, uncovering their roles in *Salix* salt stress tolerance is important for the creation of new germplasms and the provision of environmental protection in the future [44].

### SmAP2-17 may serve as a TF and play roles in salt stress tolerance

In this study, *SmAP2-17* encoding a TF protein of the SmAP2/ERF family was cloned from *S. matsudana* to determine its potential role in *Salix* resistance to salt stress. The phylogenetic tree analysis of SmAP2-17 and other AP2 subfamily proteins from maize, rice, *Arabidopsis*, and other *Salix* species showed that the SmAP2-17 protein was more similar to members of the *Salix* genus, especially SwEVM0024941 and SiEVM0034317 from *S. wilsonii* and *S. integra*, respectively (Fig. 1a). All of the members of the *Salix* genus showed 40% identity to *Arabidopsis* ANT protein, which plays roles both in plant development and abiotic stress tolerance [26, 45]. Furthermore, the multiple alignment of amino acid sequences indicated that SmAP2-17 protein had two conserved AP2 domains in the central region with characteristics similar to those of other SmAP2 proteins and *Arabidopsis* ANT (Fig. 1b). *SmAP2-17* was a ubiquitously expressed gene detected in all tissues examined, whose expression could be highly induced by salt stress (Fig. 3a). Colocalization with the nuclear marker histone H_2_B showed that the SmAP2-17 protein was localized to the nucleus (Fig. 3b). Promoter motif analysis revealed TC-rich repeats and ABRE elements, which are likely to function in defense, stress responsiveness, and abscisic acid responsiveness, respectively. Among the six homolog genes from *Salix*,* SmAP2-17* was the only gene with TC-rich repeat elements (Fig. 2). These results suggest that SmAP2-17 may serve as a TF and play an important role in salt stress tolerance.

### Analysis of salt resistance in *SmAP2-17*-overexpression transgenic *Arabidopsis*

To determine whether *SmAP2-17* is involved in regulating the salt stress response, overexpression transgenic lines were obtained and their salt tolerance abilities were studied. The results of phenotype analysis and physiological index showed that, under salt stress, the overexpression transgenic lines did not possess higher salt tolerance ability. These results implied that SmAP2-17 was not a positive regulator in the salt stress response. However, the expression level of *SmAP2-17* in T-1 and T-15 after salt stress did not support this conclusion (Fig. 4). The qRT-PCR results showed that, after salt treatment, the high levels of *SmAP2-17* expression in T-1 and T-15 fell sharply; therefore, SmAP2-17 could not work properly after salt stress (Fig. 4g). The reduced expression level of *SmAP2-17* may not be the consequence of post-transcriptional silencing because of its higher expression level, since under normal conditions, silence phenomena did not occur. Thus, we proposed that *SmAP2-17* may be the target of a salt-induced microRNA, with studies previously reporting several salt-induced microRNA genes in *Arabidopsis* [31]. Through the analysis of RNA secondary structures, we found that ath-MIR167d can bind to *SmAP2-17* mRNA to initiate mRNA degradation (Fig. S1).

To provide support for our proposition and to further clarify the function of *SmAP2-17*, another version of *SmAP2-17* with an optimized codon at the binding site was introduced into *Arabidopsis*, revealing two new overexpression transgenic lines, Op T-3 and Op T-6. Phenotype analysis and physiological index measurement provided evidence that SmAP2-17 played a positive role in salt stress tolerance and that the overexpression of *SmAP2-17* in *Arabidopsis* could lead to a higher tolerance to conditions of salt stress (Fig. 5).

To obtain the overexpression transgenic lines, the cauliflower mosaic virus (CaMV) 35S promoter was used. The 35S promoter is the most frequently used promoter, with a strong and constitutive expression pattern [46]. Although it functions as a constitutive expression pattern, the 35S promoter has variable expression patterns in different organs of *Arabidopsis thaliana* and in response to abiotic stress [46, 47]. However, the phenomena reported in our study on transgenic lines, namely that salt stress induced a sharp miRNA-mediated reduction in the expression of *SmAP2-17*, has not been previously reported.

With regards to the increased sensitivity of the T-1 and T-15 transgenic lines to salt stress, although we were unable to provide a reasonable explanation, the data of four physiological indexes and the downregulation of stress marker genes were all in line with the salt-sensitive phenotype (Fig. 4, Fig. 6b).

### SmAP2-17 may directly regulate the expression of *SOS3* and *ABI5*

Some studies have demonstrated that several AP2 TF genes enhance tolerance to salt stress through the regulation of stress/ABA-responsive genes and genes from the salt stress signaling regulatory network [11, 26, 41, 48]. To gain insights into the molecular functions of SmAP2-17 in salt stress tolerance, we detected several well-characterized stress-responsive marker genes and salt stress in transgenic lines (Fig. 6). The qRT-PCR results of gene expression showed that 13 out of the 15 genes detected were upregulated in transgenic lines. Five genes, including *RD29A*, *ABI5*, *SOS3*, *AtHKT1*, and *RBohF*, were significantly upregulated, and the expression levels of these genes were consistent with the expression levels of *SmAP2-17* in different transgenic lines and lines treated with salt stress (Fig. 6a, Fig. 4g). In T-15, which had the highest *SmAP2-17* expression level, the expression levels of five genes were also the highest. After salt stress in T15, the expression of *SmAP2-17* decreased sharply, followed by that of the marker genes mentioned above (Fig. 6b). In the Op T-3 and Op T-6 plants, salt treatment did not influence the expression level of *SmAP2-17*, and the expression of *RD29A*, *ABI5*, *SOS3*, *AtHKT1*, and *RBohF* remained at a higher level (Fig. 6c).

The expression levels of these genes were consistent with those of *SmAP2-17*, which suggested that SmAP2-17 may regulate the expression of these genes directly or indirectly. In the promoter/regulatory sequence of representative target genes of the AP2/ERF proteins, many conserved binding motifs, including GCC-box (AGCCGCC), DRE/CRT elements, and coupling elements 3-like (CE3-like) (CGCG) exist [48–50]. We detected the cis-elements in the promoter region of five genes and found that, except for the *RD29A* gene promoter, at least one or several motifs were found in the regulatory region of four other genes (Fig. 7a). The results of a dual luciferase reporter assay showed that SmAP2-17 binds to the promoter region of *SOS3* and *ABI5* and activates the expression of the downstream *LUC* gene (Fig. 7b). These results indicate that *SOS3* and *ABI5* are the direct downstream genes of SmAP2-17 in transgenic *Arabidopsis*. SOS3 is a component of the salt-overly sensitive (SOS) pathway; it could physically interact with the Thr/Ser protein kinase SOS2. Then SOS2 phosphorylated SOS1 after being recruited to the plasma membrane (PM). SOS1 is a PM Na^+^/H^+^ antiporter responsible for excluding Na^+^ from the cytosol to the apoplast and the soil solution to mitigate salt stress [51, 52]. *ABI5* encodes a member of the basic leucine zipper transcription factor family. It involves in ABA signaling during seed maturation and germination and also plays a positive role in drought and high-salinity conditions [53, 54]. By upregulating the expression levels of *SOS3* and *ABI5*, the overexpression of *SmAP2-17* in *Arabidopsis* improves the tolerance of transgenic plants to salt stress by inducing ABA signaling and regulating salt stress-related genes.

## Conclusions

In this study, *SmAP2-17* was cloned from *Salix matsudana*, and its function in salt tolerance was determined. By upregulating *SOS3* and *ABI5,* SmAP2-17 was found to play a positive role in salt stress tolerance, and the overexpression of *SmAP2-17* in *Arabidopsis* could increase the tolerance of *Arabidopsis*. Our results provide a basis for the further functional characterization of *SmAP2-17* in *Salix* and the potential use of this salt-resistant gene in the creation of new *Salix* germplasms.

## Methods

### Plant materials, growth conditions, and treatments

*Arabidopsis thaliana* ecotype *Col-0* was obtained from Arabidopsis Biological Resource Center with permission and used for the genetic transformation of the *SmAP2-17* gene. Sterilized *Arabidopsis* seeds of the *Col-0* (WT) and transgenic lines were sown on the agar medium of 1/2 Murashige and Skoog (MS) agar medium, and vernalized in the dark for 2 days at 4 °C. The seedlings were then cultured under long-day conditions (16 h light/8 h dark cycle) at 22 °C. For transgenic line screening, 25 mg/L hygromycin was added to the medium; for salt stress treatment with different concentrations of NaCl, different amounts of NaCl were dissolved into the medium. After growing for 2 weeks on medium, the seedlings were transplanted into soil and cultured in a greenhouse at 22 °C under long-day conditions. The 5-week-old plants were watered with 200 mM NaCl to mimic the salt stress treatment. After treatment for 72 h, leaf samples of *Arabidopsis* were collected and used to measure the levels of antioxidant enzyme activity and the MDA content.

The *salix matsudana* cultivar ‘9901’ was used in this study. ‘9901’ willow, a fine and widely planted variety of willow, was selected by many willow experts from Shandong Academy of Forestry. ‘9901’ willow was introduced and cultivated in the botany Garden of Nantong University and authorized for only scientific research purpose. One-year-old, 10-cm long cuttings of *Salix matsudana* ‘9901’clone were planted in Hoagland solution for 20 days to induce shoots and roots. The cuttings with shoots and roots were transferred to a solution of 150 mM NaCl and cultured for 24 h. Willow samples from different organs and root samples after salt stress were collected and used for RNA extraction.

### Cloning and sequence analysis of *SmAP2-17*

The coding sequence of *SmAP2-17* was amplified from the cDNA of *S. matsudana* using gene-specific primers (Table S2) and ligated into the pGEM-T vector through T-A cloning. Lasergene software was used to deduce the amino acid sequence of SmAP2-17. *Arabidopsis* ANT protein and other homologous proteins from *Salix purpurea*, *Populus trichocarpa*, maize, rice and other *Salix* genus were obtained by blasting against proteins datasets downloaded from the Phytozome (https://phytozome.jgi.doe.gov/pz/portal.html) and NCBI (https://www.ncbi.nlm.nih.gov/) websites. The sequence alignment of these proteins was performed using the ESPript website (https://npsa-prabi.ibcp.fr/cgi-bin/npsa_automat.pl?page=/NPSA/npsa_clustalw.html). MEGA 6.0 was used to construct a phylogenetic tree of SmAP2-17 and other plant ANT-Like proteins using the neighbor-joining (NJ) method [55]. The cis-acting elements in the promoter of *SmAP2-17* and its five homolog genes from the *Salix* genus were predicted using the online PlantCARE tool (http://bioinformatics.psb.ugent.be/webtools/plantcare/html/) and illustrated using TBtools software [56, 57]. The coding sequences and promoter sequences of *SmAP2-17* and its homologs are provided in File S1 and S2.

### Plasmid construction and transformation of *SmAP2-17* in *Arabidopsis*

The full-length coding sequence of *SmAP2-17* (File S1) amplified from pGEM-T-SmAP2-17 using specific primers listed in Table S2 were inserted into the pWM101 vector using Hieff Clone™ Plus One Step Cloning recombination Kit (Yeasen, Shanghai). The *SmAP2-17* cDNA was cloned under the control of the cauliflower mosaic virus 35S (CaMV 35S) promoter, the construct was named pWM101-35S:SmAP2-17. The optimized codon of the *SmAP2-17* CDS sequence (File S1) was synthesized by General Biology Company (Hefei, China), and the same protocol was used to obtain the pWM101-35S:Op-SmAP2-17 construct.

The pWM101-35S:SmAP2-17 and pWM101-35S:Op-SmAP2-17 plasmids were then transformed into *Agrobacterium tumefaciens* GV3101 competent cells by electroporation, and *Arabidopsis* genetic transformation was performed using the floral dip method [58]. All *SmAP2-17/Op-SmAP2-17*-overexpression transgenic seedlings were screened using 25 mg/L hygromycin to obtain positive plants. These plants were then further verified by genotyping and expression level detection.

### Subcellular localization of SmAP2-17

Using the one-step cloning method, the complete coding sequence of *SmAP2-17* amplified by PCR using specific primers was inserted into the pCAMBIA2300-35S:NEGFP vector under the control of the CaMV 35S promoter to generate an in-frame fusion construct, 35S:EGFP-SmAP2-17. As a positive nuclear marker, the cDNA of a previously characterized nuclear histone protein, H_2_B, was fused to the mCherry gene to generate 35S:H2B-mCherry. The constructs of 35S:H2B-mCherry and 35S:EGFP-SmAP2-17 were co-transformed into *Agrobacterium tumefaciens* strain GV3101 (pSoup-19) using the heat shock method. Agroinfiltration was performed as previously reported [59]. Recombination agrobacteria were suspended in 10 mM MgCl_2_ and 150 mg/l acetosyringone. The cells were allowed to stand in this medium for 3 h, and then infiltrated into the abaxial air spaces of young leaves of 2- to 4-week-old *N. benthamiana* plants using a syringe with the needle removed. The agroinfiltrated tobacco leaves after 48 h agro-infiltration were photographed using a confocal laser scanning microscope (Leica, Germany).

### RNA isolation and real-time qRT-PCR analysis

Total RNA was extracted from different organs of *S. matsudana* or *Arabidopsis* seedlings using the TaKaRa MiniBEST Plant RNA Extraction Kit (Takara, Beijing, China). Reverse transcription for the first strand cDNA synthesis was performed with 3 µg of total RNA using PrimeScript™ RT reagent Kit with gDNA Eraser (Perfect Real Time) (Takara, Beijing, China). The qRT-PCR experiments were performed on an ABI 7500 Real-Time PCR system (Applied Biosystems, USA) with TB Green™ Premix Ex Taq™ II (Tli RNaseH Plus) (Takara, Beijing, China). The actin genes *AtActin2* (*AT3G18780*) and *SpActin1* (*SapurV1A.0655s0050.1*) from *Arabidopsis* and *S. matsudana* were used as internal reference genes to normalize the data. The expression level differences between different tissues, samples, and treatments were evaluated using the 2^−ΔΔCt^ method, as previously described [60]. Three independent biological repeats were performed to ensure an accurate statistical analysis. The specific primers used are listed in Table S2.

### Dual luciferase reporter assays

To verify the interaction between SmAP2-17 and the putative AP2/ERF binding sites in the promoter sequences of five genes, including *RD29A*, *ABI5*, *SOS3*, *AtHKT1*, and *RBohF*, luciferase reporter assays were conducted in the model plant species tobacco via transient expression as previously reported [61, 62]. The PCR-amplified 2000-bp promoter sequences from five genes were cloned into pGreenII 0800-LUC vectors to obtain reporter plasmids. The cloning procedure was the same as the *SmAP2-17* CDS cloning procedure, that is, through T-A cloning and one-step recombination cloning. The construct pCAMBIA2300-35S:SmAP2-17-NEGFP was used as an effector construct, while the pCAMBIA2300-35S:NEGFP construct was used as a negative effector control. The effector/negative effector control and reporter constructs were subsequently transferred into *Agrobacterium tumefaciens* strain GV3101 (P19). The recombinant *A. tumefaciens* strains were cultured to an OD_600_ of 1.0, and strains with effector/negative effector control and reporter constructs were mixed in a 1:1 proportion. After sedimentation, the confluent bacterium was re-suspended in 10 ml of infiltration media (10 mM MgCl_2_, 0.5 M acetosyringone) to an OD_600_ of 0.2, and incubated at room temperature without shaking for 2 h. The tobacco leaves of 2- to 4-week-old plants were then infiltrated using the virus genes activated GV3101 (P19) using a needle-free syringe. After infiltration, the tobacco plants were incubated in an illuminated chamber for 48–72 h at 25 °C, a Dual-Luciferase Reporter Assay System (Promega, USA) was used to determine *LUC* and *REN* luciferase activities, according to the manufacturer’s instructions.

### Analysis of salt tolerance

Sterilized seeds from the WT, *SmAP2-17*-overexpression, and codon-optimized *SmAP2-17*-overexpression plants were sown on 1/2 MS solid medium (control) or 1/2 MS medium containing different concentrations of NaCl (75 mM, 100 mM, 150 mM) to measure the germination rate after 72 h of culture. For the root length phenotype, the seeds of the WT and codon-optimized *SmAP2-17*-overexpression plants were sown on 1/2 MS solid medium and cultured for 14 days. Then, seedlings with the same length were transferred to 1/2 MS solid medium containing 100 Mm NaCl. After 8 days, the lengths of the roots of the WT and transgenic seedlings were compared. Two-week-old seedlings of the WT, *SmAP2-17*-overexpression, and codon-optimized *SmAP2-17*-overexpression lines were transplanted into soil from 1/2 MS medium and watered for two weeks before the stress treatment experiments. For salt stress, the seedlings were irrigated with 30 mL of 200 mM NaCl solution once every two days, or with pure water as a control. After 96 h, the physiological parameters, including MDA content and levels of antioxidant enzyme activity (e.g. peroxidase (POD), superoxide dismutase (SOD), and catalase (CAT)) were measured using the corresponding kits, according to the manufacturer’s instructions (Solarbio, Beijing, CN). All experiments included three biological replicates.

## Supplementary Information

**Additional file 1:** **Table S1.** Cis-element motifs in the promoter region of *SmAP2-17* and its homologous genes.

**Additional file 2: File S1.** Protein sequences of *SmAP2-17* and its homolog genes.

**Additional file 3:** **File S2.** Promoter sequences of *SmAP2-17* and its homolog genes.

**Additional file 4:** **Table S2.** List of primers used.

**Additional file 5:** **Fig. S1.** The secondary complementary structure of *SmAP2-17* mRNA and salt-induced miRNA ath-MIR167d.

## Data Availability

Proteins and nucleotides sequences in this study were downloaded from the Phytozome (https://phytozome.jgi.doe.gov/pz/portal.html) and NCBI (https://www.ncbi.nlm.nih.gov/) websites and analyzed using ESPript (https://npsa-prabi.ibcp.fr/cgi-bin/npsa_automat.pl?page=/NPSA/npsa_clustalw.html) and PlantCARE tool (http://bioinformatics.psb.ugent.be/webtools/plantcare/html/) websites. Sequence data of proteins and promoters described in this article are available using the accession numbers listed in Supplementary File S1 and S2 respectively. The datasets supporting the conclusions of this article are included within the article and its additional files, and available from the corresponding author on reasonable request.

## Abbreviations

AP2/ERF: APETALA2/ethylene responsive factor
ORF: Open reading frame
CDS: Coding sequence
TF: Transcription factor
GFP: Green fluorescent protein
RFP: Red fluorescent protein
SOD: Superoxide dismutase
POD: Peroxidase
MDA: Malondialdehyde
CAT: Catalase
WT: Wild-type
qRT-PCR: Quantitative reverse transcription polymerase chain reaction
LUC: Luciferase
REN: Renilla
CaMV: Cauliflower mosaic virus
MS: Murashige and Skoog
