# Correction to: Overexpression of the *Salix matsudana SmAP2-17* gene improves *Arabidopsis* salinity tolerance by enhancing the expression of *SOS3* and *ABI5*

**DOI:** 10.1186/s12870-022-03552-6

**Published:** 2022-04-11

**Authors:** Yanhong Chen, Yuanhao Dai, Yixin Li, Jie Yang, Yuna Jiang, Guoyuan Liu, Chunmei Yu, Fei Zhong, Bolin Lian, Jian Zhang

**Affiliations:** Key Lab of Landscape Plant Genetics and Breeding, School of Life Science, Nantong University, Nantong, Jiangsu Province China

**Correction to: BMC Plant Biol 22, 102 (2022)**

**https://doi.org/10.1186/s12870-022-03487-y**

Following publication of the original article [1], it was noted that due to a typesetting error Fig. 3 and Fig. 6 were captured incorrectly.

The correct figures and captions have been included in this correction, and the original article has been corrected.

**Fig. 3:**
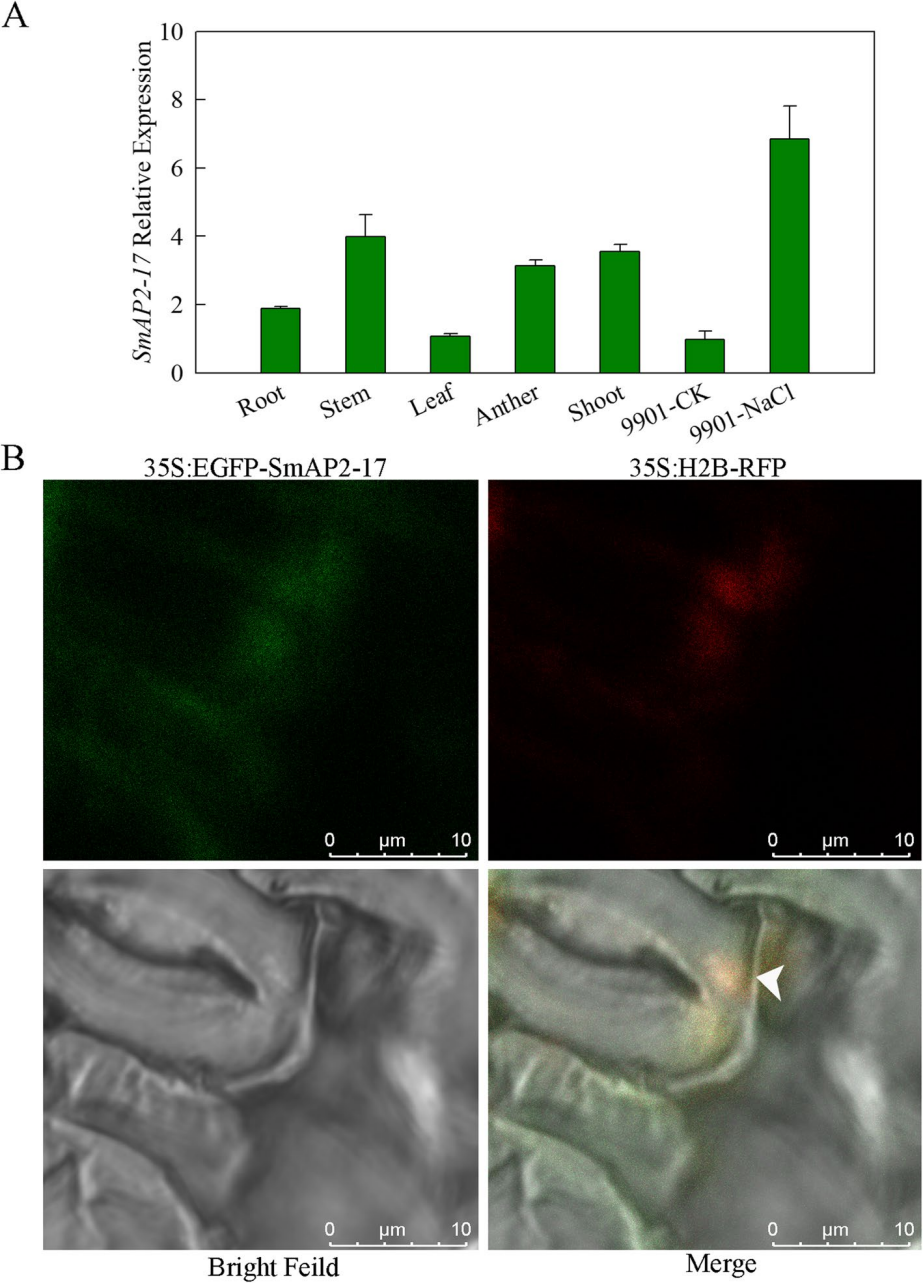
Expression pattern and subcellular localization of SmAP2-17 protein. **a** Expression patterns of *SmAP2-17* in the roots, stems, leaves, anthers, and shoots of *S. matsudana* and under salt stress were measured using qRT-PCR. **b** Subcellular localization of the SmAP2-17 protein. The 35S:EGFP-SmAP2-17 fusion construct and the nucleus localization marker 35S:H_2_B-RFP construct were co-transformed into tobacco epidermal leaves. The arrowhead indicates the merged signal (yellow) with EGFP (green) and RFP (red) co-located in the nucleus. Scale bar, 10 μm

**Fig. 6:**
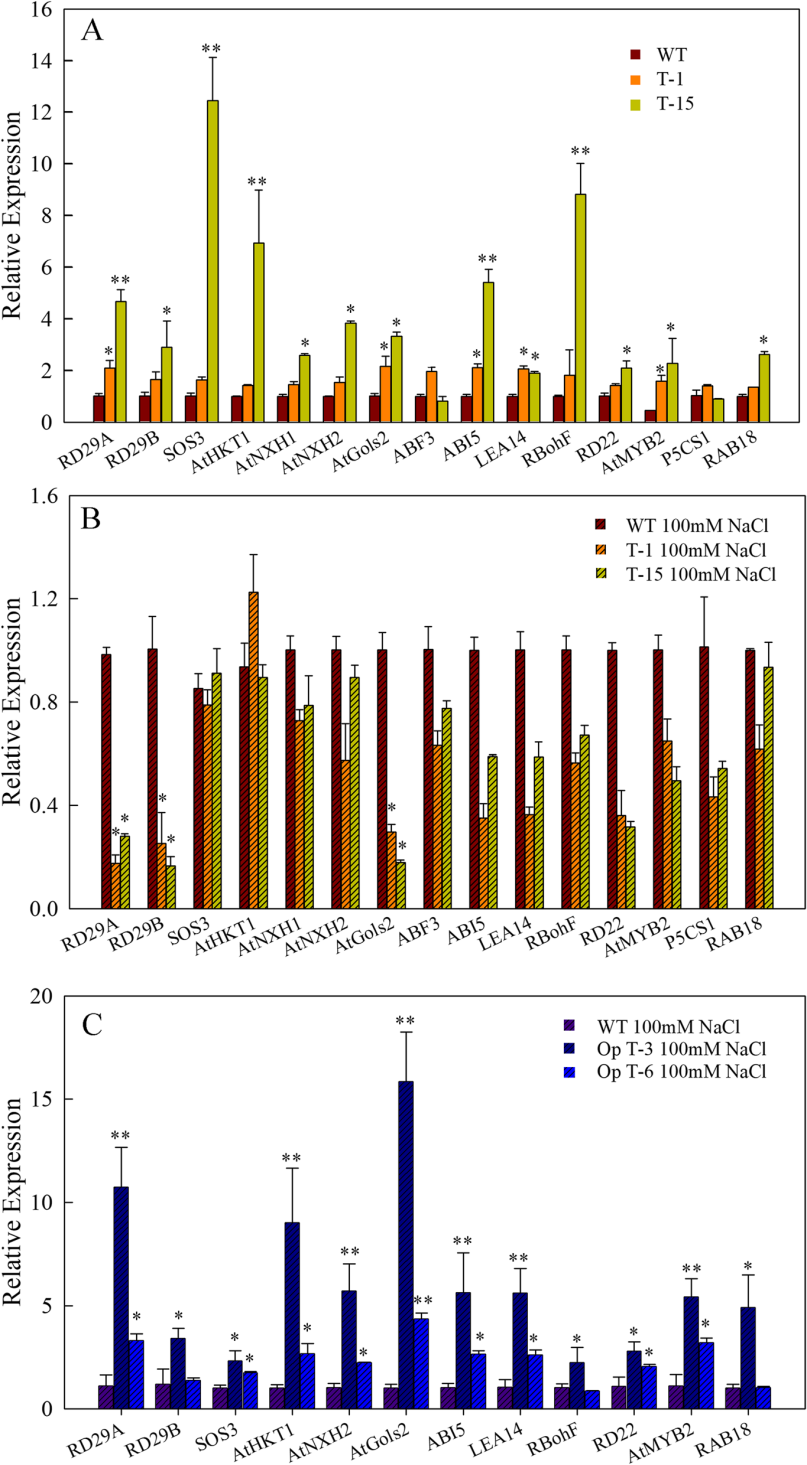
Relative expression levels of stress responsive marker genes and salinity responsive genes in WT and transgenic lines under normal conditions and after treatment with NaCl. **a** Relative expression levels of 15 genes in the WT and transgenic lines T-1 and T-15 under normal conditions. **b** Relative expression levels of 15 genes in the WT and transgenic lines T-1 and T-15 treated with 200 mM NaCl for 24 h. **c** Relative expression levels of 12 genes in the WT and transgenic lines Op T-3 and Op T-6 treated with 200 mM NaCl for 24 h. Data represent the mean ± SD of three biological replicates. *P < 0.05 and **P < 0.01 by Student’s t-test
