# Supplementary material for: Overexpression of the Salix matsudana SmAP2-17 gene improves Arabidopsis salinity tolerance by enhancing the expression of SOS3 and ABI5
Source: BMC Plant Biol. 2022 Mar 7;22:102. doi: 10.1186/s12870-022-03487-y (PMC8900321; doi:10.1186/s12870-022-03487-y)
Supplement: Supplementary file 1 — Additional file 1: Table S1. Cis-element motifs in the promoter region of SmAP2-17 and its homologous genes. [file 12870_2022_3487_MOESM1_ESM.docx]

| Gene name | Cis-acting element name | Conserved Sequence | Motif amount | Probable function |
| --- | --- | --- | --- | --- |
| SapurV1A.0608s0160 | TCA-element | CCATCTTTTT | 1 | Salicylic acid responsiveness |
| SapurV1A.0608s0160 | ARE | AAACCA | 5 | Anaerobic induction |
| SapurV1A.0608s0160 | MBS | CAACTG | 1 | Drought-inducibility |
| SmAP2-17 | TCA-element | TCAGAAGAGG | 1 | Salicylic acid Responsiveness |
| **SmAP2-17** | **TC-rich repeats** | **GTTTTCTTAC** | **1** | **Defense and stress responsiveness** |
| **SmAP2-17** | **ABRE** | **ACGTG** | **1** | **Abscisic acid responsiveness** |
| SmAP2-17 | ARE | AAACCA | 3 | Anaerobic induction |
| SmAP2-17 | TGACG-motif | TGACG | 2 | MeJA-responsiveness |
| SmAP2-17 | CGTCA-motif | CGTCA | 2 | MeJA-responsiveness |
| SmAP2-17 | MBS | CAACTG | 1 | Drought-inducibility |
| SmAP2-54EVM0057600 | ARE | AAACCA | 5 | Anaerobic induction |
| SmAP2-54EVM0057600 | MBS | CAACTG | 1 | Drought-inducibility |
| SsEVM0017996 | TATC-box | TATCCCA | 1 | Gibberellin-responsiveness |
| SsEVM0017996 | ABRE | CGTACGTGCA | 1 | Abscisic acid responsiveness |
| SsEVM0017996 | ABRE | ACGTG | 1 | Abscisic acid responsiveness |
| SsEVM0017996 | ARE | AAACCA | 5 | Anaerobic induction |
| SsEVM0017996 | MBS | CAACTG | 1 | Drought-inducibility |
| StEVM0034317 | ABRE | ACGTG | 2 | Abscisic acid responsiveness |
| StEVM0034317 | ARE | AAACCA | 2 | Anaerobic induction |
| StEVM0034317 | TGACG-motif | TGACG | 2 | MeJA-responsiveness |
| StEVM0034317 | CGTCA-motif | CGTCA | 2 | MeJA-responsiveness |
| StEVM0034317 | MBS | CAACTG | 1 | Drought-inducibility |
| SwEVM0024941 | ARE | AAACCA | 7 | Anaerobic induction |
| SwEVM0024941 | MBS | CAACTG | 2 | Drought-inducibility |

Table S1. The list of Cis-elements motifs in the promoter region of *SmAP2-17* and its homologs genes.
