# Supplementary material for: Overexpression of the Salix matsudana SmAP2-17 gene improves Arabidopsis salinity tolerance by enhancing the expression of SOS3 and ABI5
Source: BMC Plant Biol. 2022 Mar 7;22:102. doi: 10.1186/s12870-022-03487-y (PMC8900321; doi:10.1186/s12870-022-03487-y)
Supplement: Supplementary file 4 — Additional file 4: Table S2. List of primers used. [file 12870_2022_3487_MOESM4_ESM.docx]

Table S2 primer list

| Primer name | Primer sequence（5’→3’） | | | |
| --- | --- | --- | --- | --- |
| SmAP2-17 T-A Cloning |  | | | |
| SmAP2-17-F | AGAAATGAAGTCCATCAACAATG | | | |
| SmAP2-17-R | ACACTAATCCTAGGCGTCTGTC | | | |
| SmAP2-17 Subcellular location/Luc Assay Effector | | | | |
| p2300-SmAP2-17-F | GACGAGCTGTACAAGGGATCCAGAAATGAAGTCCATCAACAATG | | | |
| p2300-SmAP2-17-R | CTGCAGGTCGACTCTAGATCACACTAATCCTAGGCGTCTGTC | | | |
| SmAP2-17 Overexpression | | | | |
| pWM101-SmAP2-17-F | TCGAGCTTTCGCGAGCTCGGTACCAGAAATGAAGTCCATCAACAATG | | | |
| pWM101-SmAP2-17-R | GCATGCCTGCAGGTCGACTCTAGAACACTAATCCTAGGCGTCTGTC | | | |
| SmAP2-17 RNAi | | | | |
| TCTAGAGGCGCGCCTGGTACC AGTAGAGAAGAATCTGTACGACAAACTA***T***CAAAGCAACCATGATGATCACATTCCGTTATCTATTTTTTGGTTGCTTTG***C***TAGTTTGTCGCATTGGCTCTTCTTACT GTCGACGCAGAAGATCTTCCC  Underline：p1300-35S-C vector sequence; red：21nt siRNA and its complementary sequence; other：mir390a gene scaffold | | | | |
| amiRNA-F | TCTAGAGGCGCGCCTGGTACCAGTAGAGAAGAATCTGTA | | | |
| amiRNA-R | GGGAAGATCTTCTGCGTCGACAGTAAGAAGAGCCAATG | | | |
| Downstream gene promoter TA cloning | | | | |
| TA RD29A F  TA RD29A R  TA SOS3 F  TA SOS3 R  TA HKT1 F  TA HKT1 R  TA ABI5 F | AGATTTGGGGTTTTGCTT TTGAAT  AAGATTTTTTTCTTTCCA ATAGAA  GCAAGTTTTTTTTTTTTT CATTTT  ATTCGTTTTCGCTGGTTT TACTTC  GCTTGAACTAGGAATCCCTAGATC  TTTAGTTCTCGAGTCGGTTTAAGC  TAATCTTTTTTATTTTTGTTTTTA | | | |
| TA ABI5 R  TA RbohF F | CTGAAAATGAAATCTGTGTGTCTA  ACTATGGTCTATGGACTTGGTG | | | |
| TA RbohF R | TCGGAATTCAAAGAGTTGGTTG | | | |
| Downstream gene promoter subcloning into pGreenII 0800-LUC | | | | |
| RD29A- LUC F | TATAGGGCGAATTGGGTACCAGATTTGGGGTTTTGCTTTTGAAT | | | |
| RD29A- LUC R | TGTTTTTGGCGTCTTCCATGAAGATTTTTTTCTTTCCAATAGAA | | | |
| SOS3- LUC F | TATAGGGCGAATTGGGTACCGCAAGTTTTTTTTTTTTTCATTTT | | | |
| SOS3- LUC R | TGTTTTTGGCGTCTTCCATGATTCGTTTTCGCTGGTTTTACTTC | | | |
| HKT1- LUC F | TATAGGGCGAATTGGGTACCGCTTGAACTAGGAATCCCTAGATC | | | |
| HKT1- LUC R | TGTTTTTGGCGTCTTCCATGTTTAGTTCTCGAGTCGGTTTAAGC | | | |
| ABI5- LUC F | TATAGGGCGAATTGGGTACCTAATCTTTTTTATTTTTGTTTTTA | | | |
| ABI5- LUC R | TGTTTTTGGCGTCTTCCATGCTGAAAATGAAATCTGTGTGTCTA | | | |
| RbohF - LUC F  RbohF - LUC R | TATAGGGCGAATTGGGTACCACTATGGTCTATGGACTTGGTG  TGTTTTTGGCGTCTTCCATGTCGGAATTCAAAGAGTTGGTTG | | | |
| Q-PCR Primers |  | | | |
| AtActin-F | | GCCATCCAAGCTGTTCTCTC |  |  |
| AtActin-R | | GCTCGTAGTCAACAGCAACAA |  |  |
| QAtNXH1-F | | AAGAGCAGCGTTCGTCTTTC |  |  |
| QAtNXH1-R | | TGAGACCAGACCACCAAATCAC |  |  |
| QAtNXH2-F | | TCAATCCACATTCCGCTCCTC |  |  |
| QAtNXH2-R | | AAACCTCGAAGGCTGTTTGG |  |  |
| QAtCHX16-F | | ACGGTTTGCCCTTTTGGAAC |  |  |
| QAtCHX16-R | | TGAAATCGCGGTCACAGAAC |  |  |
| QAtMPK4-F | | AAGCAGACGCATCACAGTTG |  |  |
| QAtMPK4-R | | TTGAACGGCCTCACACATAC |  |  |
| QAtMPK6-F | | TAAGTTCCCGACAGTGCATCC |  |  |
| QAtMPK6-R | | TGGGCCAATGCGTCTAAAAC |  |  |
| QAtSOS1-F | | AGAACACAGAGGTCTCATGAGC |  |  |
| QAtSOS1-R | | TGCCGAAAATGCTGAGTTGC |  |  |
| QAtSOS2-F | | TGACAGGCGACAGGATTTTG |  |  |
| QAtSOS2-R | | TGAGTTCGCTACAGCCTCAATG |  |  |
| QAtSOS3-F | | CGCCGGTCCATGAAAAAGTC |  |  |
| QAtSOS3-R | | TTCGGATTCGTGAAGAAGCG |  |  |
| QRAB18-F | | GTCTTACTGCTGAAGGTTCGTCTG |  |  |
| QRAB18-R | | ATCCAAGATGCTGCGGTTTAGG |  |  |
| QUBQ10-F | | CGGAAAGCAGTTGGAGGATGG |  |  |
| QUBQ10-R | | CGGAGCCTGAGAACAAGATGAAG |  |  |
| QABI5-F | | AATGGAGGAGGTGGTGGTGAG |  |  |
| QABI5-R | | CTGCTGCTGCTTGTTGTTGATTG |  |  |
| QRD29A-F | | GTGACGACGAAGTTACCTATCTCC |  |  |
| QRD29A-R | | TCTCCGCCACATAATCTCTACCC |  |  |
| QRD29B-F | | CAGACAGAGGAGAGAGCAGAGAG |  |  |
| QRD29B-R | | CTTCACCACCAGGAGCAAACG |  |  |
| QLEA14-F | | GATTTCTTCTGATCGACAAAACCTA |  |  |
| QLEA14-R | | AGCAAACCCAACTTATTACATTACG |  |  |
| QDREB2A-F | | GACCTAAATGGCGACGATGT |  |  |
| QDREB2A-R | | TCGAGCTGAAACGGAGGTAT |  |  |
| QRbohF-F | | CTGCGGTTTCGCCATTC |  |  |
| QRbohF-R | | TGTTTCGTCGGCTCTG |  |  |
| QRbohD-F | | ATTACAAGCACCAAACCAG |  |  |
| QRbohD-R | | TGCCAAGCCATAACATCA |  |  |
| QATGolS2-F | | GACGAGTCTCTTGATTACAAGAATGTT |  |  |
| QATGolS2-R | | AAACTGCTGAAGTGTCTGTTGC |  |  |
| QRD22-F | | CGATTCGTCTTCCTCTGAT |  |  |
| QRD22-R | | TACTCCGCCTTTACCTACT |  |  |
| QMYB2-F | | GTTAGAAGAGGCAACATCACT |  |  |
| Sp Actin1-Q-F | | GTCAAGTTCTTTGCTTTCCTCC |  |  |
| Sp Actin1-Q-R | | CATCACAATCACTCTCCGACTA | |  |
| QSmAP2-17-F | | ACGAGGCGGATTGGAAAATG | |  |
| QSmAP2-17-R  OpQSmAP2-17-F  OpSmAP2-17-R | | TTCAGGGGACAGTGATAATGCC  AAAAGCGATGGCAGTCTGTG  GTGCCAATATTTGCGCCAAC | |  |
|  | |  | |  |
