# Supplementary figures and images for: Overexpression of the Salix matsudana SmAP2-17 gene improves Arabidopsis salinity tolerance by enhancing the expression of SOS3 and ABI5
Source: BMC Plant Biol. 2022 Mar 7;22:102. doi: 10.1186/s12870-022-03487-y (PMC8900321; doi:10.1186/s12870-022-03487-y)

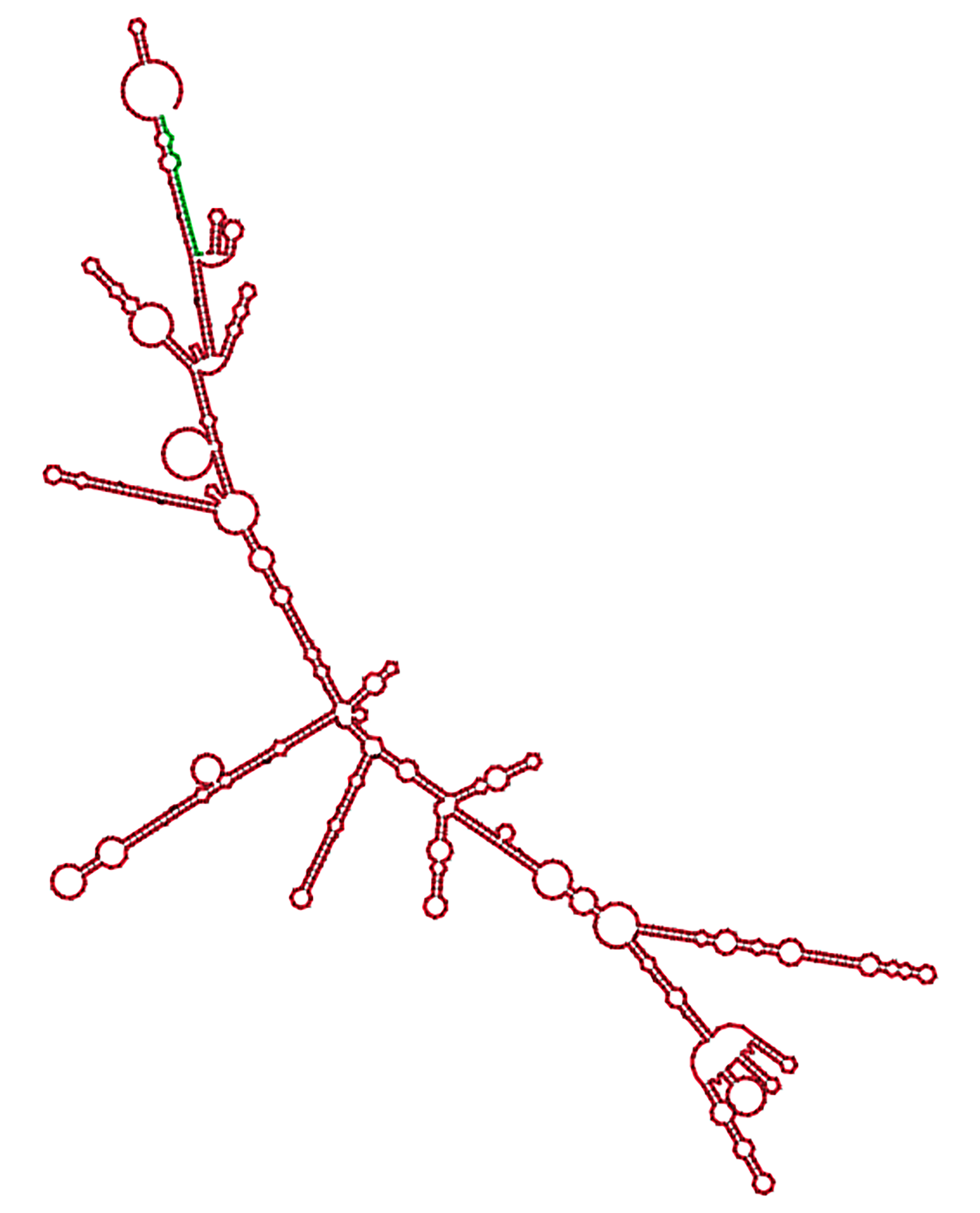

Supplement: Supplementary file 5 — Additional file 5: Fig. S1. The secondary complementary structure of SmAP2-17 mRNA and salt-induced miRNA ath-MIR167d. [file 12870_2022_3487_MOESM5_ESM.tif]
